# A Combined Extract from *Dioscorea bulbifera* and *Zingiber officinale* Mitigates PM_2.5_-Induced Respiratory Damage by NF-κB/TGF-β1 Pathway

**DOI:** 10.3390/antiox13121572

**Published:** 2024-12-20

**Authors:** In Young Kim, Hyo Lim Lee, Hye Ji Choi, Yeong Hyeon Ju, Yu Mi Heo, Hwa Rang Na, Dong Yeol Lee, Won Min Jeong, Ho Jin Heo

**Affiliations:** 1 Division of Applied Life Science (BK21), Institute of Agriculture and Life Science, Gyeongsang National University, Jinju 52828, Republic of Korea; inzero331@gnu.ac.kr (I.Y.K.); gyfla059@gnu.ac.kr (H.L.L.); hjchoi0820@gnu.ac.kr (H.J.C.); ju8172001@gnu.ac.kr (Y.H.J.); yumi@gnu.ac.kr (Y.M.H.); hrna@gnu.ac.kr (H.R.N.); 2 Research & Development Team, Gyeongnam Anti-Aging Research Institute, Sancheong 52215, Republic of Korea; dylee1984@gari.or.kr (D.Y.L.); jwm5618@gari.or.kr (W.M.J.)

**Keywords:** *Dioscorea bulbifera*, *Zingiber officinale*, respiratory dysfunction, oxidative stress, inflammation, fibrosis

## Abstract

This research evaluated the protective role of a combined extract of *Dioscorea bulbifera* and *Zingiber officinale* (DBZO) against respiratory dysfunction caused by particulate matter (PM_2.5_) exposure in BALB/c mice. The bioactive compounds identified in the DBZO are catechin, astragalin, 6-gingerol, 8-gingerol, and 6-shogaol. DBZO ameliorated cell viability and reactive oxygen species (ROS) production in PM_2.5_-stimulated A549 and RPMI 2650 cells. In addition, it significantly alleviated respiratory dysfunction in BALB/c mice exposed to PM_2.5_. DBZO improved the antioxidant systems in lung tissues by modulating malondialdehyde (MDA) content, as well as levels of reduced glutathione (GSH) and superoxide dismutase (SOD). Likewise, DBZO restored mitochondrial dysfunction by improving ROS levels, mitochondrial membrane potential, and ATP production. Moreover, DBZO modulated the levels of neutrophils, eosinophils, monocytes, and lymphocytes (specifically CD4^+^, CD8^+^, and CD4^+^IL-4^+^ T cells) in blood and IgE levels in serum. DBZO was shown to regulate the c-Jun N-terminal kinase (JNK) pathway, nuclear factor kappa B (NF-κB) pathway, and transforming growth factor β (TGF-β)/suppressor of mothers against decapentaplegic (Smad) pathway. Histopathological observation indicated that DBZO mitigates the increase in alveolar septal thickness. These findings indicate that DBZO is a promising natural agent for improving respiratory health.

## 1. Introduction

Air quality has deteriorated worldwide due to urbanization and population growth, which has created public health challenges [1]. Among various air pollutants, particulate matter (PM) is particularly recognized for its heightened risk to human health compared to other pollutants [2]. Various studies have reported that exposure to PM can trigger respiratory diseases such as chronic obstructive pulmonary disease (COPD), idiopathic pulmonary fibrosis (IPF), and asthma [3,4]. PM is categorized based on aerodynamic diameter into PM_10_ (particles ≤ 10 µm), PM_2.5_ (particles ≤ 2.5 µm), and PM_0.1_ (particles ≤ 0.1 µm) [5]. Of these, PM_2.5_ is particularly concerning due to its high specific surface area relative to its mass which allows it to adsorb harmful substances such as zinc (Zn), iron (Fe), and polycyclic aromatic hydrocarbons (PAHs) from the atmosphere and enter the body, leading to oxidative stress and inflammatory responses [6]. Inorganic elements attached to PM_2.5_ that enter the body generate reactive oxygen species (ROS) through the Fenton reaction, inducing oxidative stress, while PAHs are also known to penetrate the body and produce metabolic byproducts that contribute to cellular injury and increased ROS production [7,8]. The overproduction of ROS leads to the depletion of the endogenous antioxidant system, such as reduced glutathione (GSH) and superoxide dismutase (SOD), resulting in increased oxidative stress [9]. It promotes the infiltration of inflammatory cells into the respiratory system and the secretion of chemokines and cytokines, eventually triggering the activation of nuclear factor kappa B (NF-κB) proteins that drive inflammatory responses [10,11]. Furthermore, chronic inflammation has been reported to promote pulmonary fibrosis through the activation of the transforming growth factor-β (TGF-β)/suppressor of mothers against decapentaplegic (Smad) pathway [12]. Against this backdrop, numerous studies are being conducted to explore the relationship between oxidative stress and inflammatory responses and to develop strategies for their mitigation, with natural antioxidants emerging as a primary approach [13,14,15].

Natural products, rich in antioxidants, have been used for centuries in health and treatment, and play a crucial role in traditional medicine [16]. Among these, *Dioscorea bulbifera* (*D. bulbifera*) and *Zingiber officinale* (*Z. officinale*) are plants traditionally used in Asian medicine for their therapeutic efficacy [17,18]. *D. bulbifera*, also known as air potato, is a member of the *Dioscoreaceae* family and has been traditionally used to treat conditions such as laryngopharyngitis and chest pain [19,20]. It contains various polyphenols and flavonoids, including catechin, quercetin, and kaempferol, which have been reported to exhibit respiratory protective effects through their antioxidant and anti-inflammatory activities [21,22,23]. Similarly, *Z. officinale*, or ginger, is a member of the *Zingiberaceae* family and has been used to alleviate respiratory diseases such as colds and sore throats [18]. Its biological activity is attributed to bioactive compounds such as gingerol, shogaol, paradol, and zingerone [18]. In particular, gingerol and shogaol have been extensively studied for their antioxidative and anti-inflammatory characteristics, as well as their protective roles in the respiratory system [24]. Previous research has demonstrated that combining herbs with similar biological activities, such as antioxidant and anti-inflammatory properties, can more effectively alleviate pathological symptoms by targeting multiple metabolic pathways than using a single herb [25,26]. This effect is believed to arise from the synergistic interaction among the various bioactive components [25,26]. Thus, this study aimed to investigate the protective properties of a combined extract of *D. bulbifera* and *Z. officinale* (DBZO), each traditionally used in therapies for treating respiratory diseases, in BALB/c mice with PM_2.5_-induced respiratory dysfunction.

## 2. Materials and Methods

### 2.1. Preparation of PM_2.5_

PM_2.5_ was purchased from Powder Technology Incorporated (Nominal 0–3 micron arizona test dust, Arden Hills, MN, USA), with an average particle size of 1.06 µm. This particle was used as PM_2.5_ in this study. The components of PM_2.5_ used in this study were identified as Al, Fe, Mg, Mn, Ba, Zn, and Cu [27]. It was dissolved in purified water and used for cell and animal experiments.

### 2.2. Preparation of DBZO

DBZO extract powder was offered by the Gyeongnam Anti-Aging Research Institute on 23 February 2024. *D. bulbifera* was purchased from local market (Jinju, Republic of Korea) as a fresh sample. It was cut into pieces and dried in a drying oven (JOURI-Q, KEC, Seoul, Republic of Korea) at 45 °C for 48 h. *Z. officinale* was purchased as a dried sample from Donghae (Seoul, Republic of Korea). Next, the dried *D. bulbifera* and *Z. officinale* were combined at a 9:1 ratio. The mixture was then subjected to reflux extraction with 50% ethanol at 90 °C for 4 h. Afterward, it was filtered, concentrated, freeze-dried, and stored at −80 °C until further use.

### 2.3. Ultra-Performance Liquid Chromatography-Quadrupole Time-of-Flight Mass Spectrometry (UPLC-Q-TOF/MS)

The DBZO used for the analysis was prepared by dissolving it in 50% methanol. The analysis was conducted using UPLC-Q-TOF/MS (Waters, Milford, MA, USA) equipped with an Acquity UPLC BEH C_18_ column (2.1 mm × 100 mm, 1.7 μm; Waters). The column temperature was set to 40 °C and the mobile phase was composed of water containing 0.1% formic acid (solvent A) and acetonitrile containing 0.1% formic acid (solvent B), with a flow rate of 0.35 mL/min. For chromatographic separation, solvent B was initially maintained at 1% for 1 min, then linearly increased to 100% from 1 to 8 min. Solvent B was held at 100% from 8 to 9 min, decreased to 1% from 9 to 9.5 min, and finally maintained at 1% from 9.5 to 12 min. Subsequently, the column eluents were detected using the Q-TOF-MS with positive electrospray ionization (ESI) mode. Mass spectrometry conditions were applied as follows: capillary voltage, 3 kV; ion source temperature, 100 °C; desolvation temperature, 400 °C; cone gas, 30 L/h; desolvation gas, 800 L/h; collision energy, 20–40 eV; and mass range, 50–1500 *m*/*z*.

### 2.4. Cytoprotective Effects of DBZO Against PM_2.5_-Induced A549 and RPMI 2650 Cells

#### 2.4.1. Cell Cultures

A549 cells (KCLB, Seoul, Republic of Korea) and RPMI 2650 cells (ATCC, Manassas, VA, USA) were cultured in RPMI1640 and MEM media, respectively, each supplemented with 10% fetal bovine serum and 1% penicillin/streptomycin. All cells were cultured in an incubator maintained at 37 °C and 5% CO_2_.

#### 2.4.2. Cell Viability

A549 and RPMI 2650 cells were plated in a 96-well plate at a density of 1 × 10^4^ cells/well and incubated for 24 h. Then, cells were pre-treated for 30 min with phosphate-buffered saline (PBS), vitamin C (100 μg/mL), or DBZO (at concentrations of 10, 20, 50, 100, and 200 μg/mL). Vitamin C was used as a positive control in this study due to its extensively reported cellular protective effects against oxidative stress [28]. The dose of DBZO was selected based on previous studies reporting non-cytotoxic yet biologically effective ranges [20,29]. After 30 min, cultured cells were treated with PBS or PM_2.5_ (100 µg/mL) and cultured for 24 h. The dose of PM_2.5_ was selected based on previous studies (Figures S1 and S2). Following this, the MTT solution was added to each well for 3 h. The medium was then replaced with dimethyl sulfoxide after it was suctioned. Absorbance was measured at 570 nm (determination wavelength) and 655 nm (reference wavelength) using a microplate reader (Epoch2, BioTek Instruments Inc., Winooski, VT, USA).

#### 2.4.3. Intracellular Oxidative Stress

A549 and RPMI 2650 cells were plated in a 96-well black plate at a density of 1 × 10^4^ cells/well and cultured for 24 h. Then, cells were pre-treated for 30 min with PBS, vitamin C (100 μg/mL), or DBZO (at concentrations of 10, 20, 50, 100, and 200 μg/mL). Vitamin C was used as a positive control in this study due to its extensively reported cellular protective effects against oxidative stress [28]. The dose of DBZO was selected based on previous studies reporting non-cytotoxic yet biologically effective ranges [20,29]. After 30 min, cultured cells were treated with PBS or PM_2.5_ (100 µg/mL) and cultured for 24 h. The dose of PM_2.5_ was selected based on previous studies (Figures S1 and S2). Following this, the DCFH-DA solution was added to each well for 50 min. Fluorescence was measured at 485 nm (excitation wavelength) and 535 nm (emission wavelength) using a fluorometer (Infinite F200, TECAN, Mannedorf, Switzerland).

### 2.5. Animals

BALB/c mice (6 weeks, male) were purchased from Samtako (Osan, Republic of Korea) and housed under controlled environmental conditions, including a 12 h light/dark cycle, a temperature of 22 ± 2 °C, and a humidity of 50 ± 5%. The mice were randomly divided into five groups (*n* = 15 per group) as follows: normal control (NC, clean air exposure + drinking water), normal sample (NS, clean air exposure + DBZO 100 mg/kg of body weight), PM_2.5_ (PM_2.5_ exposure + drinking water), DBZO50 (PM_2.5_ exposure + DBZO 50 mg/kg of body weight), and DBZO100 (PM_2.5_ exposure + DBZO 100 mg/kg of body weight). After an adaptation period of 1 week, the animals were orally administered either clean water or DBZO before exposure to the air in the chamber. Then, the whole bodies of the mice were exposed in a chamber to clean air or PM_2.5_ at a concentration of 500 μg/m^3^ for 5 h/day and 5 days/week for 12 weeks. The dose of PM_2.5_ was selected based on previous studies and WHO guidelines [30,31,32,33]. After 12 weeks, mice were dissected to collect blood and lung samples for subsequent analyses. All animal experiments were performed with the permission of the Institutional Animal Care and Use Committee (IACUC) of Gyeongsang National University (GNU-240108-M0002, date of approval: 8 January 2024).

### 2.6. Effects of DBZO Against PM_2.5_-Induced Antioxidant System Dysfunction

#### 2.6.1. Malondialdehyde (MDA) Contents

The lung tissues obtained from the mice were homogenized with PBS. The homogenate was centrifuged at 4 °C, 2356× *g* for 10 min to obtain the supernatants. The supernatants were heated in a water bath set to 95 °C with 1% phosphoric acid and 0.67% thiobarbituric acid. The reactants were measured at 532 nm using a spectrophotometer (UV-1800, Shimadzu, Tokyo, Japan).

#### 2.6.2. Reduced GSH Levels

The lung tissues obtained from the mice were homogenized with 10 mM phosphate buffer, including 1 mM ethylenediamine tetraacetic acid (EDTA), and centrifuged at 4 °C and 10,000× *g* for 15 min. The supernatants were mixed with 5% metaphosphoric acid and re-centrifuged at 4 °C and 2000× *g* for 2 min to obtain supernatant. Subsequently, it was mixed with 0.26 M Tris-HCl (pH 7.5), 0.65 N NaOH, and 1 mg/mL o-phthalaldehyde. Fluorescence was measured at 360 nm (excitation wavelength) and 430 nm (emission wavelength) using a fluorometer (Infinite F200, TECAN).

#### 2.6.3. SOD Levels

The lung tissues obtained from the mice were homogenized with PBS and centrifuged at 4 °C and 400× *g* for 10 min to obtain the pellet. Subsequently, it was mixed with an extraction buffer and then reacted on ice for 30 min. The reactants were centrifuged at 4 °C and 10,000× *g* for 10 min. Afterward, the SOD kit (Dojindo Molecular Tech., Rockville, MD, USA) was used according to the manufacturer’s instructions.

### 2.7. Effects of DBZO Against PM_2.5_-Induced Mitochondrial Dysfunction

#### 2.7.1. Extraction of Mitochondria from Lung Tissue

The lung tissues obtained from the mice were homogenized with mitochondrial isolation (MI) buffer [0.1% bovine serum albumin, 20 mM HEPES sodium salt, 75 mM sucrose, and 215 mM mannitol] containing 1 mM ethylene glycol-bis(β-aminoethyl ether)-N,N,N′,N′-tetraacetic acid (EGTA). The homogenate was centrifuged at 4 °C and 1300× *g* for 5 min to obtain the supernatant. Subsequently, it was re-centrifuged at 4 °C and 13,000× *g* for 10 min to obtain the pellets. Next, the pellet was treated with MI buffer containing 0.1% digitonin and incubated on ice for 5 min. After that, MI buffer containing 1 mM EGTA was added to the reactant and centrifuged at 4 °C and 13,000× *g* for 15 min. The obtained pellet was re-centrifuged at 4 °C and 10,000× *g* for 10 min after adding the MI buffer. Finally, the collected pellet was mixed with MI buffer to obtain the samples for analysis.

#### 2.7.2. ROS Levels

To measure mitochondrial ROS levels, mitochondrial extracts from the lung tissues were mixed with a DCFH-DA solution dissolved in respiration buffer (500 μM EGTA, 1 mM MgCl_2_, 2 mM KH_2_PO_4_, 2.5 mM malate, 5 mM pyruvate, 20 mM HEPES, and 125 mM KCl). Fluorescence was measured at 485 nm (excitation wavelength) and 535 nm (emission wavelength) using a fluorometer (Infinite F200, TECAN).

#### 2.7.3. Mitochondrial Membrane Potential

To evaluate mitochondrial membrane potential levels, mitochondrial extracts from lung tissues were mixed in 1,1′,3,3′-Tetraethyl-5,5′,6,6′-tetrachloroimidacarbocyanine iodide (JC-1) solution dissolved in assay buffer (5 mM pyruvate and 5 mM malate). Fluorescence was measured at 535 nm (excitation wavelength) and 590 nm (emission wavelength) using a fluorometer (Infinite F200, TECAN).

#### 2.7.4. ATP Contents

The ATP kit (Promega Corporation, Madison, WI, USA) was used to measure the ATP contents in mitochondrial extracts from lung tissues according to the manufacturer’s instructions. Luminescence was measured using a luminometer (Glomax^®^, Promega Corporation).

### 2.8. White Blood Cells (WBC) Differential Counting

After dissection, whole blood was obtained from the abdominal vena cava and placed in the ethylenediaminetetraacetic acid dipotassium salt dihydrate (K_2_EDTA) tube. Then, WBC (neutrophils, lymphocytes, monocytes, eosinophils, and basophils) were analyzed using SYSMEX XN-V (Sysmex Corporation, Kobe, Japan).

### 2.9. Flow Cytometry

All materials used in flow cytometry were obtained from BD Bioscience (Franklin Lakes, NJ, USA) except for the PerCP-Cy5.5-conjugated CD8a and PE-conjugated IL-4, which were obtained from BioLegend (San Diego, CA, USA). Additionally, all washing processes were conducted at 4 °C and 126× *g* for 5 min.

After dissection, whole blood was immediately collected from the abdominal vena cava and placed in the heparin tube. The collected blood was stained with BV786-conjugated CD3e (#564379), PE-Cy7-conjugated CD4 (#552775), and PerCP-Cy5.5-conjugated CD8a (#100734) at 4 °C for 30 min. Next, the lysing solution (#349202) was added to lyse the red blood cells, followed by washing the pellet using the stain buffer (#554657). It was then fixed and permeabilized through the fixation/permeabilization solution kit (#554715) at 4 °C for 20 min. Following this, intracellular cytokines were incubated with PE-conjugated IL-4 (#504104) at 4 °C for 30 min, and then washed using the wash buffer provided in the fix/perm kit. Finally, the samples were suspended in the stain buffer and analyzed using the FACSLyric (BD Bioscience). Data were analyzed using FlowJo (version 10.10.0, BD Biosciences).

### 2.10. Enzyme-Linked Immunosorbent Assay (ELISA)

The serum was obtained by centrifuging whole blood collected in heparin tubes at 4 °C, 10,000× *g* for 15 min. The IgE level in the serum was determined using ELISA kit (Abcam, Cambridge, UK) according to the manufacturer’s instructions. Absorbance was measured at 450 nm using a microplate reader (Epoch 2, BioTek Instruments Inc.).

### 2.11. Hematoxylin-Eosin (H&E) Staining

To observe the morphological changes, the left lungs of mice were fixed in 10% formalin. After dehydration, the lung tissues were embedded in paraffin and cut into sagittal sections (4 μm). The H&E-stained slides were scanned with a Motic EasyScan Pro 6 (Motic, Hong Kong, China), and the alveolar space was analyzed using ImageJ (version 1.54d, National Institutes of Health, Bethesda, MD, USA).

### 2.12. Western Blot

The lung tissues obtained from the mice were homogenized with ProtinEx™ Animal cell/tissue (GeneAll Biotechnology, Seoul, Republic of Korea) containing 1% protease inhibitor Cocktail kit tissue 2 perfect (Quartett, Berlin, Germany) and centrifuged at 4 °C and 15,928× *g* for 10 min. The obtained supernatants were used in a Bradford assay (Bio-Rad, Hercules, CA, USA) and mixed with 4X sample buffer (Bio-Rad). Using sodium dodecyl-sulfate polyacrylamide gel electrophoresis, proteins were separated, then transferred onto a polyvinylidene difluoride membrane (Millipore, Burlington, MA, USA). The membrane was treated with 5% skimmed milk at room temperature for 1 h and then incubated with the primary antibody (1:1000) at 4 °C for 12 h. Following three washes, the membrane was incubated with a secondary antibody (1:3000) at room temperature for 1 h. In the final step, the proteins were detected using an ECL ottimo (Translab, Daejeon, Republic of Korea) and iBright CL1500 (Thermo Fisher Scientific, Walthman, MA, USA). The band density was quantified using ImageJ (version 1.54d, National Institutes of Health). The information on the primary and secondary antibodies used is summarized in Table 1.

### 2.13. Statistics Analysis

All data were shown as mean ± standard deviation. Statistical comparison was performed with one-way analysis of variance (ANOVA). Duncan’s multiple range test was used for multiple group comparisons, and Student’s *t*-test was used for single comparisons.

## 3. Results

### 3.1. Identification of Bioactive Compounds

**Figure 1:**
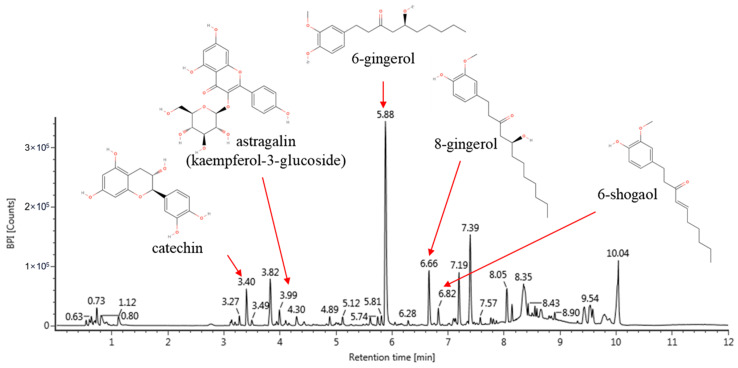
Ultra-performance liquid chromatography quadrupole time-of-flight mass spectrometry (UPLC-Q-TOF/MS) chromatogram of a combined extract from *Dioscorea bulbifera* and *Zingiber officinale* (DBZO).

The results of identifying the bioactive compounds of DBZO through UPLC-Q-TOF/MS are as follows: catechin (retention time (RT), 3.40 min; adduct ion, 291; fragments; 139 and 273), astragalin (kaempferol-3-glucoside) (RT, 3.99 min; adduct ion, 449; fragments; 257, 269, and 287), 6-gingerol (RT, 5.88 min; adduct ion, 317; fragments, 115, 117, and 145), 8-gingerol (RT, 6.66 min; adduct ion, 345; fragments, 115 and 145), and 6-shogaol (RT, 6.82 min; adduct ion, 277; fragments, 94, 122, 137, and 177) (Figure 1 and Table 2) [34,35,36].

### 3.2. Cytoprotective Effects of DBZO Against PM_2.5_-Induced A549 and RPMI 2650 Cells

#### 3.2.1. Cell Viability

**Figure 2:**
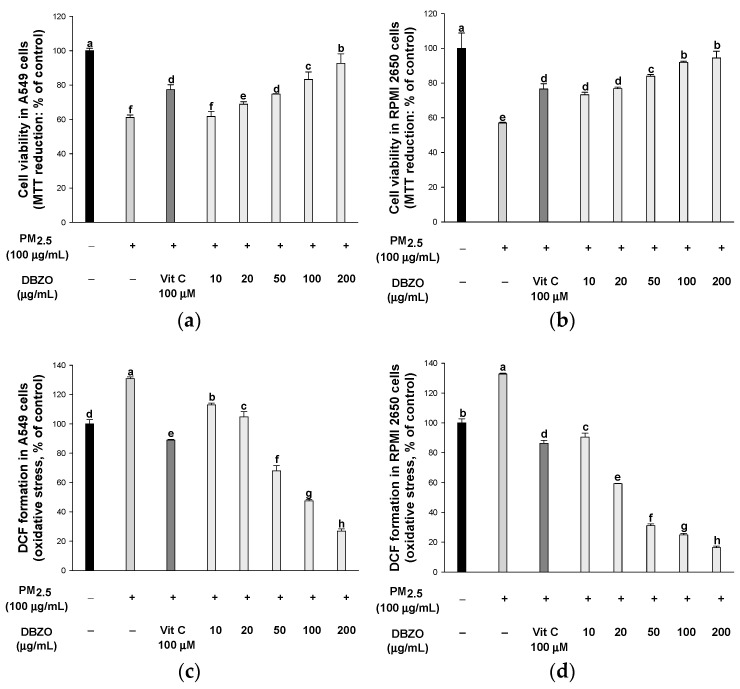
Effects of DBZO of particulate matter (PM_2.5_)-stimulated A549 and RPMI 2650 cells. Cell viability in (**a**) A549 and (**b**) RPMI 2650 cells and intracellular oxidative stress levels in (**c**) A549 and (**d**) RPMI 2650 cells. The results are presented as mean ± SD (*n* = 3). Data were statistically considered at *p* < 0.05, and different small letters represent the statistical differences.

The PM_2.5_ treatment reduced cell viability to 61.11% in A549 cells compared to the control (100%). However, the treatment with vitamin C positive control showed an increase in cell viability to 77.38%. Similarly, the treatment with DBZO showed an increase in cell viability, with 61.66% at 10 μg/mL, 68.80% at 20 μg/mL, 74.69% at 50 μg/mL, 83.28% at 100 μg/mL, and 92.61% at 200 μg/mL (Figure 2a).

The PM_2.5_ treatment reduced cell viability to 56.98% in RPMI 2650 cells compared to the control (100%). However, the treatment with vitamin C positive control showed an increase in cell viability to 76.61%. Similarly, the treatment with DBZO showed an increase in cell viability, with 73.25% at 10 μg/mL, 76.77% at 20 μg/mL, 83.93% at 50 μg/mL, 91.90% at 100 μg/mL, and 94.44% at 200 μg/mL (Figure 2b).

#### 3.2.2. Intracellular Oxidative Stress

The PM_2.5_ treatment increased ROS production to 130.87% in A549 cells compared to the control (100%). However, the treatment with vitamin C positive control reduced ROS production to 88.92%. Similarly, the treatment with DBZO reduced ROS production, with 113.02% at 10 μg/mL, 104.70% at 20 μg/mL, 67.93% at 50 μg/mL, 47.57% at 100 μg/mL, and 26.93% at 200 μg/mL (Figure 2c).

The PM_2.5_ treatment increased ROS production to 132.70% in RPMI 2650 cells compared to the control (100%). However, the treatment with vitamin C positive control reduced ROS production to 86.32%. Similarly, the treatment with DBZO reduced ROS production, with 90.38% at 10 μg/mL, 59.36% at 20 μg/mL, 31.12% at 50 μg/mL, 24.90% at 100 μg/mL, and 16.32% at 200 μg/mL (Figure 2d).

### 3.3. Effects of DBZO Against PM_2.5_-Induced Antioxidant System Dysfunction

#### 3.3.1. MDA Contents

**Figure 3:**
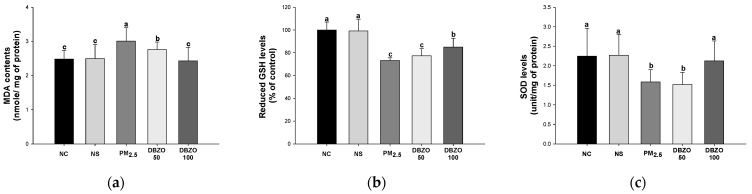
Effects of DBZO on antioxidant system in the lung tissues of PM_2.5_-exposed BALB/c mice. (**a**) Malondialdehyde (MDA) contents, (**b**) reduced glutathione (GSH) levels, and (**c**) superoxide dismutase (SOD) levels. The results are presented as mean ± SD (*n* = 5). Data were statistically considered at *p* < 0.05, and different small letters represent the statistical differences.

The PM_2.5_ group (3.01 nmol/mg of protein) showed an increase in MDA contents compared to the NC group (2.48 nmol/mg of protein). However, the DBZO100 group (2.43 nmol/mg of protein) demonstrated a significant reduction in MDA contents compared to the PM_2.5_ group (Figure 3a).

#### 3.3.2. Reduced GSH Levels

The PM_2.5_ group (73.13%) showed a decrease in reduced GSH levels compared to the NC group (100.00%). However, the DBZO100 group (85.02%) exhibited a significant increase in reduced GSH levels compared to the PM_2.5_ group (Figure 3b).

#### 3.3.3. SOD Levels

The PM_2.5_ group (1.59 unit/mg of protein) showed a reduction in SOD levels compared to the NC group (2.25 unit/mg of protein). However, the DBZO100 group (2.13 unit/mg of protein) showed a significant increase in SOD levels compared to the PM_2.5_ group (Figure 3c).

### 3.4. Effects of DBZO Against PM_2.5_-Induced Mitochondrial Dysfunction

#### 3.4.1. ROS Levels

**Figure 4:**
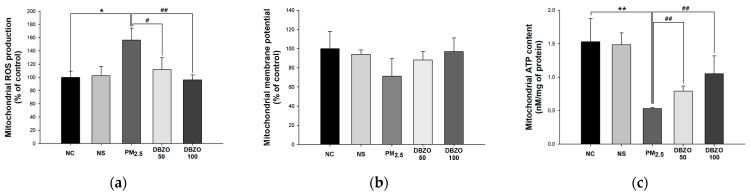
Effects of DBZO on mitochondrial function in lung tissues of PM_2.5_-exposed BALB/c mice. (**a**) Mitochondrial reactive oxygen species (ROS) production, (**b**) mitochondrial membrane potential, and (**c**) mitochondrial ATP content. The results are presented as mean ± SD (*n* = 4). Data are statistically represented with * = significantly different from the NC group, and # = significantly different from the PM_2.5_ group; * and # = *p* < 0.05 and ** and ## = *p* < 0.01.

The PM_2.5_ group (156.20%, *p* = 0.031) showed an increase in ROS levels compared to the NC group (100.00%). However, the DBZO100 group (96.23%, *p* = 0.001) demonstrated a reduction in ROS levels compared to the PM_2.5_ group (Figure 4a).

#### 3.4.2. Mitochondrial Membrane Potential

The PM_2.5_ group (71.31%, *p* = 0.066) showed a reduction in mitochondrial membrane potential compared to the NC group (100.00%). However, the DBZO100 group (96.83%, *p* = 0.069) showed an increase in mitochondrial membrane potential compared to the PM_2.5_ group. However, there was no significant difference between the groups (Figure 4b).

#### 3.4.3. ATP Content

The PM_2.5_ group (0.53 nM/mg of protein, *p* = 0.001) showed a decrease in ATP content compared to the NC group (1.53 nM/mg of protein). However, the DBZO100 group (1.05 nM/mg of protein, *p* = 0.008) exhibited an increase in ATP content compared to the PM_2.5_ group (Figure 4c).

### 3.5. Effects of DBZO Against PM_2.5_-Induced Hematological and Biochemical Changes

#### 3.5.1. WBC Differential Counting

The PM_2.5_ group (4.0460 k/µL) showed an increase in total WBC count compared to the NC group (1.6380 k/µL). However, the DBZO100 group (2.8740 k/µL) demonstrated a significant reduction in total WBC count compared to the PM_2.5_ group.

Furthermore, the PM_2.5_ group exhibited elevated levels of neutrophils (0.9327 k/µL), lymphocytes (2.8743 k/µL), and eosinophils (0.1955 k/µL) compared to the NC group (0.3464 k/µL, 1.2477 k/µL, and 0.0078 k/µL, respectively). However, the DBZO100 group (0.5902 k/µL, 2.2140 k/µL, and 0.0258 k/µL, respectively) significantly reduced these levels compared to the PM_2.5_ group. Whereas monocytes and basophil counts showed no significant differences among the NC (0.0298 k/µL and 0.0062 k/µL), PM_2.5_ (0.0396 k/µL and 0.0040 k/µL), and DBZO100 groups (0.0402 k/µL and 0.0037 k/µL) (Table 3).

#### 3.5.2. Flow Cytometry

**Figure 5:**
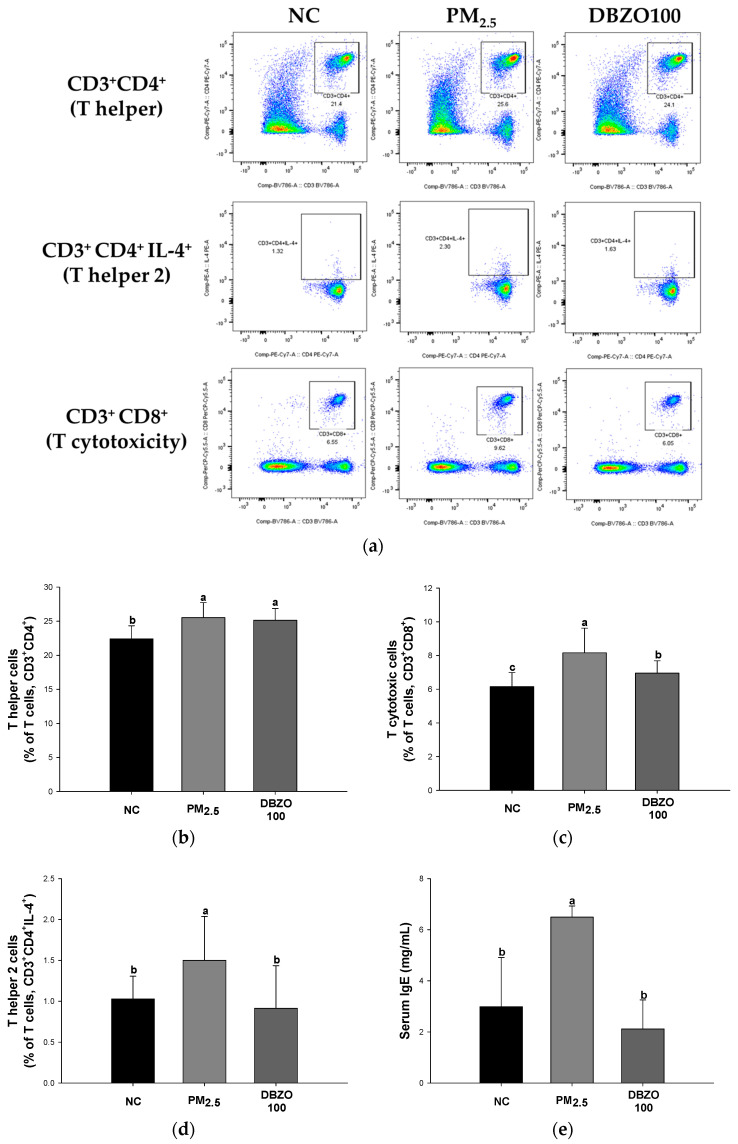
Effects of DBZO on inflammatory cells in whole blood and immunoglobulin E (IgE) levels in serum of PM_2.5_-exposed BALB/c mice. (**a**) Flow cytometry plots, frequency of (**b**) CD3^+^CD4^+^ T cells, (**c**) CD3^+^CD8^+^ T cells, (**d**) CD3^+^CD4^+^IL-4^+^ T cells in whole blood, and (**e**) IgE levels in serum. The results are presented as mean ± SD (**b**–**d**, *n* = 5; e, *n* = 3). Data were statistically considered at *p* < 0.05, and different small letters represent the statistical differences.

The PM_2.5_ group showed increased levels of T cells, including T helper cells (25.52% of T cells), T cytotoxic cells (8.16% of T cells), and T helper 2 cells (1.50% of T helper cells), compared to the NC group (22.4%, 6.15%, and 1.03% of T cells, respectively). In contrast, the DBZO100 group (25.14%, 6.95%, and 0.91% of T cells, respectively) significantly downregulated these T cell levels compared to the PM_2.5_ group (Figure 5a–d).

#### 3.5.3. IgE Analysis Using ELISA

The PM_2.5_ group (6.49 mg/mL) showed an increased IgE levels in serum compared to the NC group (2.99 mg/mL). In contrast, the DBZO100 group (2.12 mg/mL) demonstrated a significantly reduced IgE levels in serum compared to the PM_2.5_ group (Figure 5e).

### 3.6. Effects of DBZO Against PM_2.5_-Induced Histopathological Changes

**Figure 6:**
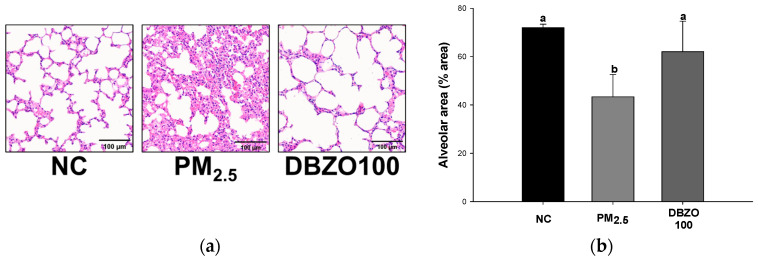
Effects of DBZO on alveolar size in lung tissues of PM_2.5_-exposed BALB/c mice. (**a**) Histopathological sections and (**b**) alveolar area. The results are presented as mean ± SD (*n* = 3). Data were statistically considered at *p* < 0.05, and different small letters represent the statistical differences.

A pathological examination of the lung tissue revealed that the NC group exhibited a normal lung architecture with no pathological changes observed (Figure 6a). In contrast, the PM_2.5_ group demonstrated marked histopathological alterations, including alveolar septal thickening due to inflammation and fibrosis, resulting in the collapse of normal alveolar spaces. The DBZO100 group, however, exhibited reduced alveolar septal thickening compared to the PM_2.5_ group, maintaining an alveolar structure similar to that of the NC group. In this regard, quantification of lung structural damage by alveolar area measurements showed that the alveolar area in the PM_2.5_ group (43.35%) increased compared to the NC group (71.96%), whereas a significant reduction in the alveolar area was observed in the DBZO100 group (62.03%), suggesting a protective effect of DBZO on lung tissue (Figure 6b).

### 3.7. Effect of DBZO Against PM_2.5_-Induced Pulmonary Inflammation-Related Factors

**Figure 7:**
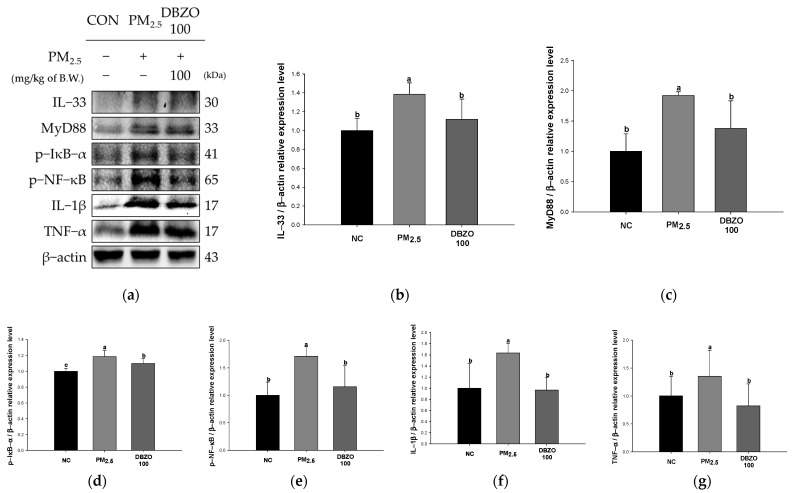
Effects of DBZO on inflammation-related protein expression levels in lung tissues of PM_2.5_-exposed BALB/c mice. (**a**) Western blot images, protein expression levels of (**b**) IL-33, (**c**) MyD88, (**d**) p-IκB-α, (**e**) p-NF-κB, (**f**) IL-1β, and (**g**) TNF-α. The results are presented as mean ± SD (*n* = 3). Data were statistically considered at *p* < 0.05, and different small letters represent the statistical differences.

The PM_2.5_ group showed a significant upregulation in the expression levels of IL-33 (1.39), MyD88 (1.92), p-IκB-α (1.19), p-NF-κB (1.71), IL-1β (1.64), and TNF-α (1.36) compared to the NC group (1.00). In contrast, the DBZO100 group demonstrated a significant downregulation in the expression levels of IL-33 (1.12), MyD88 (1.38), p-IκB-α (1.10), p-NF-κB (1.16), IL-1β (0.97), and TNF-α (0.83) compared to the PM_2.5_ group (Figure 7).

### 3.8. Effect of DBZO Against PM_2.5_-Induced Pulmonary Apoptosis-Related Factors

**Figure 8:**
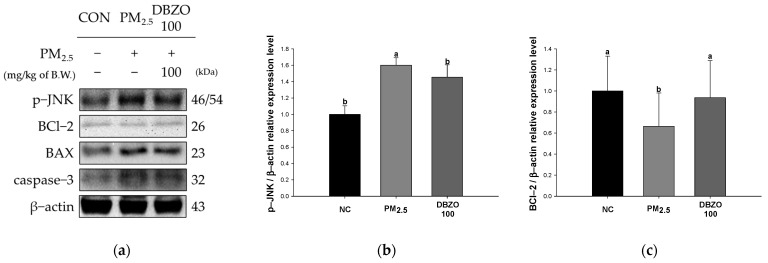

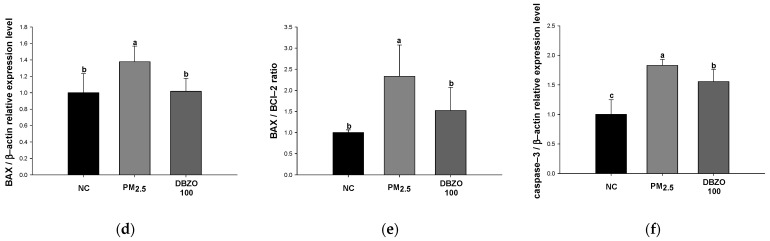
Effects of DBZO on apoptosis-related protein expression levels in lung tissues of PM_2.5_-exposed BALB/c mice. (**a**) Western blot images, protein expression levels of (**b**) p-JNK, (**c**) BCl-2, (**d**) BAX, (**e**) BAX/BCl-2 ratio, and (**f**) caspase-3. The results are presented as mean ± SD (*n* = 3). Data were statistically considered at *p* < 0.05, and different small letters represent the statistical differences.

The PM_2.5_ group showed a significant downregulation in the expression level of BCl-2 (0.67) and a significant upregulation in the expression levels of p-JNK (1.60), BAX (1.38), BAX/BCl-2 ratio (2.34), and caspase-3 (1.83) compared to the NC group (1.00). In contrast, the DBZO100 group demonstrated a significant regulation of expression levels for p-JNK (1.46), BCl-2 (0.94), BAX (1.02), BAX/BCl-2 ratio (1.52), and caspase-3 (1.56) compared to the PM_2.5_ group (Figure 8).

### 3.9. Effect of DBZO Against PM_2.5_-Induced Pulmonary Fibrosis-Related Factors

**Figure 9:**
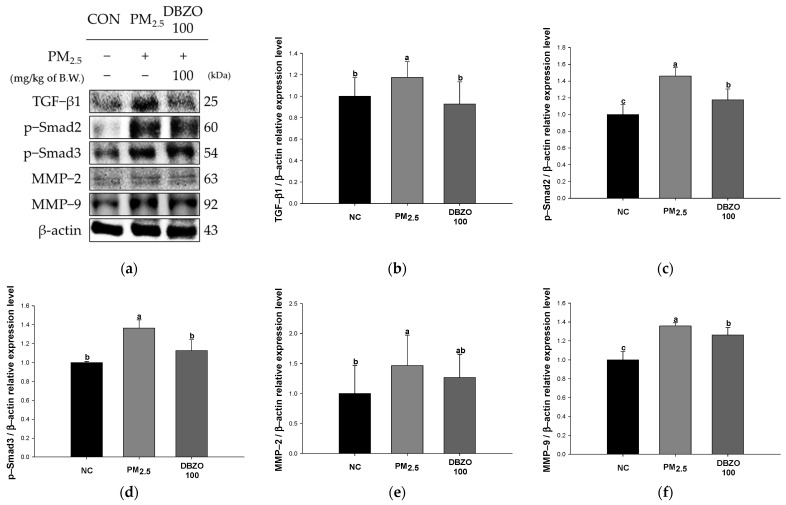
Effects of DBZO on pulmonary fibrosis-related protein expression levels in lung tissues of PM_2.5_-exposed BALB/c mice. (**a**) Western blot images, protein expression levels of (**b**) TGF-β1, (**c**) p-Smad2, (**d**) p-Samd3, (**e**) MMP-2, and (**f**) MMP-9. The results are presented as mean ± SD (*n* = 3). Data were statistically considered at *p* < 0.05, and different small letters represent the statistical differences.

The PM_2.5_ group showed a significant upregulation in the expression levels of TGF-β1 (1.18), p-Smad2 (1.46), p-Smad3 (1.36), MMP-2 (1.47), and MMP-9 (1.36) compared to the NC group (1.00). In contrast, the DBZO100 group demonstrated a significant downregulation in the expression levels of TGF-β1 (0.93), p-Smad2 (1.17), p-Smad3 (1.13), MMP-2 (1.27), and MMP-9 (1.26) compared to the PM_2.5_ group (Figure 9).

## 4. Discussion

Air pollution has emerged as a global public health issue, leading to a growing emphasis on research into chronic respiratory diseases [37]. The pathogenesis of these diseases involves a complex interplay of factors such as oxidative stress, mitochondrial dysfunction, and inflammation [3]. Therefore, natural products are being highlighted as therapeutic strategies due to their pharmacological effects, which arise from acting on various targets and simultaneously modulating multiple signaling pathways [38]. In this context, this study examined the protective role of DBZO in mitigating respiratory system damage caused by PM_2.5_ exposure.

*D. bulbifera* and *Z. officinale* are known for their various biological properties and have a long history of use in traditional medicine [17,18]. To further explore their potential, the present study analyzed DBZO, a combined extract of these two substances, using UPLC-Q-TOF/MS, which led to the identification of five phytochemicals: catechin, astragalin (kaempferol-3-glucoside), 6-gingerol, 8-gingerol, and 6-shogaol (Figure 1 and Table 2) [34,35,36]. Catechin is a representative bioactive compound reported in *D. bulbifera* [17]. Similarly, 6-gingerol, 8-gingerol, and 6-shogaol are representative bioactive compounds reported in *Z. officinale* [36]. However, astragalin identified through UPLC-Q-TOF/MS in this study has not been previously reported in the root extracts of *D. bulbifera* and *Z. officinale*. Nevertheless, *D. bulbifera* has been reported to contain kaempferol and its glycosides [17]. In this regard, *D. bulbifera* was extracted under the same conditions as DBZO and analyzed using UPLC-Q-TOF/MS, resulting in the confirmation of astragalin (Figure S3). Catechins and astragalin share a flavonoid backbone and have been reported to possess numerous biological activities, including antioxidant, anti-inflammatory, and anti-diabetic effects [39]. In particular, astragalin has been reported to exhibit protective effects against respiratory diseases such as asthma, COPD, and acute lung injury (ALI) by modulating various signaling pathways, including the nuclear factor erythroid 2-related factor 2 (Nrf2)/heme oxygenase-1 (HO-1), NF-κB, and mitogen-activated protein kinase (MAPK) pathways, thereby suppressing inflammation and oxidative stress [40]. Additionally, gingerol and shogaol, recognized components in *Z. officinale*, exhibit antioxidant and anti-inflammatory properties attributed to their structural features, including alkyl chains and double bonds, and confer various health benefits such as enhanced respiratory health, improved digestion, strengthened immune function, and promoted cardiovascular health [41]. Similarly, secondary metabolites in plants, such as flavonoids, phenolic acids, and fatty acids, are widely found in natural products and have been reported to confer various health advantages by effectively scavenging ROS through their unique structural properties [16,22,39]. Therefore, to assess the protective effects of DBZO on the respiratory system, an in vitro study was conducted using A549 (lung epithelial cells) and RPMI 2650 (nasal epithelial cells) to confirm its protective effects (Figure 2). The epithelial cells in the respiratory system serve as the primary defense against external damage, and this impairment is therefore regarded as a critical factor determining disease severity [42]. In this context, DBZO suggests a positive influence on respiratory health by preventing damage to the respiratory epithelial cells caused by exposure to PM. Therefore, an in vivo study was subsequently conducted using a mouse model chronically exposed to PM_2_._5_ to further assess the protective effects of DBZO on the respiratory system.

Oxidative stress is regarded as a primary contributor to the initiation and progression of respiratory diseases [43]. It arises from an imbalance between a diminished antioxidant defense system and increased ROS production in the body [9]. SOD is an initial response element in the antioxidant defense system in the body that catalyzes the reaction that converts O_2_^·−^ into H_2_O_2_, thereby preventing damage to essential biological structures such as DNA, proteins, and cell membranes, and protecting cells from oxidative stress [44]. Meanwhile, GSH is known for its powerful antioxidant properties in removing oxidative stress-causing substances such as OH· and O_2_, as well as aiding in the elimination of hydrogen peroxide [9]. However, PM_2.5_ contains various metal compounds such as heavy metals, carbonaceous materials, and PAHs, which are reported to trigger oxidative stress by increasing the production of ROS and compromising the innate antioxidant system in the respiratory system [6]. Consequently, this increases in oxidative stress levels in the body induces protein and lipid peroxidation, leading to elevated levels of MDA, a biomarker for chronic respiratory diseases such as COPD and asthma [45]. In this regard, catechin, a principal bioactive compound of DBZO, has been reported to mediate both a direct antioxidant mechanism by scavenging reactive radicals and an indirect antioxidant effect by inducing antioxidant enzymes such as catalase, SOD, and reduced GSH, or by inhibiting proteins that promote oxidation such as cyclooxygenase and inducible nitric oxide synthase [46]. In addition, another bioactive compound of DBZO, 6-gingerol, has been shown to regulate the levels of GSH, SOD, and MDA in mice exposed to oxidative stress caused by chlorpyrifos [47]. Consistent with these findings, lung tissue from the group treated with DBZO in this study showed modulated levels of GSH, SOD, and MDA compared to the PM_2.5_ group, indicating a recovery from PM-induced oxidative stress (Figure 3). These results indicate that DBZO could help safeguard the respiratory system from oxidative stress caused by PM exposure.

Mitochondria, the primary site of ROS production, are significantly more susceptible to oxidative stress than other cellular components, and their damage is reported to induce various physiological and pathological processes, including respiratory diseases [9,48]. Generally, the mitochondrial respiratory chain maintains a balance between ROS production and elimination, but PM_2.5_ disrupts this equilibrium, resulting in excessive ROS generation [49]. It can damage mitochondrial structural proteins and membrane potential, ultimately impairing energy metabolism and causing cellular damage [48]. Santos et al. reported that catechins regulate the function of Complex I of the respiratory chain, thereby modulating ATP synthesis capacity in MRC-5 fibroblasts with amiodarone-induced mitochondrial dysfunction [50]. Similarly, Han et al. noted that 6-gingerol exhibits cardioprotective effects by improving mitochondrial membrane damage and edema in a mouse model of cardiac toxicity induced by As_2_O_3_ [51]. Likewise, in this study, the lung tissue of the group administrated with DBZO exhibited regulated levels of ROS, mitochondrial membrane proteins, and ATP compared to the PM_2.5_ group (Figure 4). Additionally, the increased ROS resulting from oxidative stress and mitochondrial damage can activate the JNK pathway and further exacerbate oxidative stress [43]. JNK plays a crucial role in apoptosis by modulating BCl-2 family proteins, inhibiting BCl-2, an anti-apoptotic protein, while promoting the activation of BAX, a pro-apoptotic protein on the mitochondrial outer membrane, which subsequently increases membrane permeability [52]. Ultimately, this cascade of events leads to apoptosis by activating caspase-3 [53]. Cho et al. noted that astragalin inhibited the expression of p-JNK protein in BEAS-2B cells stimulated to H_2_O_2_ and concentration-dependently suppressed caspase-3 activity in BEAS-2B cells stimulated to lipopolysaccharide [53]. Furthermore, Han et al. reported that pretreatment of 6-shogaol reduced BAX and caspase-3 in ultraviolet A-stimulated human dermal fibroblast cells in a dose-dependent manner [54]. Similarly, in the lungs of the DBZO-administered group in this study, the expression of apoptosis-related proteins was improved compared to the PM_2.5_ group (Figure 8). These findings indicate that DBZO, which contains various bioactive compounds, may protect the respiratory system from PM_2.5_ exposure by protecting mitochondrial function and regulating the expression of apoptosis-related proteins.

Furthermore, increased ROS production leads to a complex inflammatory response, including the release of inflammatory cytokines and the infiltration of inflammatory cells, thereby accelerating the pathogenesis of respiratory diseases [11,42]. Specifically, excessive ROS production and a depleted antioxidant system induced by PM_2.5_ result in oxidative stress, which mediates the release of IL-33 from pulmonary epithelial cells [42,55]. The released IL-33 activates various immune cells, including neutrophils, eosinophils, basophils, and T cells, a process reported to drive the pathology of pulmonary diseases, including COPD, IPF, and asthma [56]. The activated inflammatory cells then infiltrate lung tissue through chemotaxis and secrete various cytokines and chemokines that amplify the inflammatory response [11]. Moreover, activated T cells stimulate B cells to promote the secretion of immunoglobulins such as IgE, thus sustaining the inflammatory response [57]. 6-Gingerol has been observed to exhibit anti-inflammatory and antioxidant effects by reducing neutrophil accumulation and regulating the NF-κB pathway in a ventilator-induced lung injury mouse model [58]. Astragalin has been reported to significantly reduce the numbers of eosinophils, neutrophils, basophils, and macrophages in the bronchoalveolar lavage fluid of ovalbumin (OVA)-induced mice and to suppress IgE elevation in the NC/Nga mouse model [59,60]. Similarly, this analysis demonstrated that the counts of inflammatory cells and levels of IgE in both blood and serum were altered in the DBZO group in comparison to the PM_2.5_ group (Figure 5 and Table 3). Additionally, released IL-33 is known to bind to its receptor and mediate downstream signaling through the MyD88 protein [56]. Activated MyD88 phosphorylates IκB proteins, facilitating the nuclear translocation of NF-κB, activating the transcription of pro-inflammatory cytokine genes, and leading to increased secretion of TNF-α and IL-1 [11,56]. The secretion of these cytokines induces the recruitment and activation of inflammatory cells, thereby sustaining and amplifying the inflammatory response and promoting the pathogenic process [61]. Catechin has been shown to inhibit thymic stromal lymphopoietin (TSLP), a protein with a role similar to IL-33, in human nasal epithelial cells (HNEpC), in turn suppressing the NF-κB pathway [62]. In addition, ginger extract rich in gingerol and shogaol has been reported to have shown the effect of improving clinical symptoms of the disease with the regulation of IL-33 expression in experimental autoimmune encephalomyelitis mouse models [63]. In this context, the lung tissue of the DBZO group in the present study showed a significant attenuation of the NF-κB pathway induced by IL-33 compared to the PM_2.5_ group (Figure 7). This suggests that DBZO may protect the respiratory system from PM_2.5_ exposure by inhibiting inflammatory cell infiltration, immunoglobulin secretion, and the expression of inflammation-related proteins.

Chronic exposure to PM_2.5_ induces persistent inflammation and promotes pulmonary fibrosis characterized by diffuse alveolar damage, epithelial cell phenotype changes, and fibroblast proliferation [6,64]. Notably, PM_2.5_ increases the expression of TGF-β1 in lung tissue and induces the phosphorylation of Smad2/3, mediating the classical TGF-β/Smad pathway involved in fibrosis development [6,12]. It induces the activation of fibroblasts and myofibroblasts, leading to an increase in the secretion of extracellular matrix (ECM) components and promoting the progression of fibrosis [65]. Additionally, TGF-β1 regulates the expression of epithelial-to-mesenchymal transition (EMT)-related genes, including MMP-2 and MMP-9, which promote fibrosis [66]. MMP-2 and MMP-9, categorized as gelatinases, degrade various ECM molecules to activate the EMT process, with MMP-9 playing a pivotal role in promoting sustained tissue remodeling by inducing the re-expression of TGF-β1, thereby accelerating ECM accumulation and tissue stiffening, which drives fibrosis progression [66,67]. Wang et al. reported that green tea catechins effectively mitigate liver fibrosis in CCl_4_-induced mice by inhibiting the expression of TGF-β, p-Smad2, MMP-2, and MMP-9 [68]. Similarly, Cho et al. reported that astragalin inhibits airway epithelial fibrosis in H_2_O_2_-exposed BEAS-2B cells and OVA-induced mice [60]. Similarly, Liu et al. reported that 6-gingerol, a functional component of *Z. officinale*, reduces the transcription of fibrosis-related factors such as α-SMA in lung fibroblasts treated with TGF-β1 and has shown effectiveness in reducing inflammation and fibrosis in a bleomycin-induced pulmonary fibrosis mouse model [69]. In this context, the present study revealed that the lung tissue of the DBZO group had a significantly reduced expression of fibrosis-related proteins compared to the PM_2.5_ group (Figure 9). Additionally, histological observations were conducted to examine the impact of the anti-inflammatory and anti-fibrotic properties of DBZO on lung structure and found that DBZO effectively inhibited pulmonary dysfunction and fibrotic changes (Figure 6). In conclusion, these findings indicate that DBZO possesses anti-inflammatory and anti-fibrotic effects, suggesting its potential as a candidate for improving respiratory health.

## 5. Conclusions

This study indicates that DBZO significantly ameliorates PM_2.5_-induced respiratory dysfunction by regulating oxidative stress and inflammation. DBZO suppressed the production of ROS triggered by PM_2.5_ and mitigated oxidative stress by regulating the levels of antioxidant markers. DBZO has been shown to prevent mitochondrial dysfunction and regulate JNK pathway, thereby inhibiting apoptosis. Moreover, it has been demonstrated to alleviate levels of inflammatory cells and IgE and to reduce the inflammatory response by modulating the NF-κB pathway. Furthermore, DBZO contributed to the anti-fibrotic process in the respiratory system by regulating the TGF-β1/Smad pathway. These effects were confirmed by histopathological observation. The protective effect of DBZO against PM_2.5_-induced respiratory damage is considered to be due to its bioactive compounds such as phytochemicals such as catechin, astragalin, 6-gingerol, 8-gingerol, and 6-shogaol. In conclusion, this study suggests that DBZO can be used as an ingredient in functional foods that improve oxidative stress and inflammation to prevent respiratory dysfunction.

## Figures and Tables

**Table 1:** Details of primary and secondary antibodies utilized in this study.

Antibody	Catalog No.	Manufacturer
Anti-mouse IgG	AP124P	Millipore (Billerica)
Anti-rabbit IgG	#7074	Cell Signaling Tech (Danvers, MA, USA)
β-actin	sc-69879	Santa Cruz Biotech (Dallas, TX, USA)
B-cell leukemia/lymphoma 2 (BCl-2)	sc-7382	Santa Cruz Biotech (Dallas)
BCl-2 associated X (BAX)	sc-7480	Santa Cruz Biotech (Dallas)
caspase-3	sc-56053	Santa Cruz Biotech (Dallas)
Interleukin 1β (IL-1β)	sc-515598	Santa Cruz Biotech (Dallas)
IL-33	sc-517600	Santa Cruz Biotech (Dallas)
Matrix metalloproteinase (MMP) -2	sc-13595	Santa Cruz Biotech (Dallas)
MMP-9	sc-13520	Santa Cruz Biotech (Dallas)
Myeloid differentiation primary response 88 (MyD88)	sc-74532	Santa Cruz Biotech (Dallas)
Phospho-c-Jun N-terminal kinases (p-JNK)	sc-6254	Santa Cruz Biotech (Dallas)
p-NF-κB inhibitor α (p-IκB-α)	sc-8404	Santa Cruz Biotech (Dallas)
p-NF-κB	sc-136548	Santa Cruz Biotech (Dallas)
p-Smad2	#3108	Cell Signaling Tech (Danvers)
p-Smad3	sc-517575	Santa Cruz Biotech (Dallas)
TGF-β1	sc-130348	Santa Cruz Biotech (Dallas)
Tumor necrosis factor α (TNF-α)	sc-33639	Santa Cruz Biotech (Dallas)

**Table 2:** Physiological compounds identified in DBZO using UPLC-Q-TOF/MS analysis.

No.	Retention Time	Proposed Compound	(+) ESI-MS (*m*/*z*)	Fragments (*m*/*z*)
1	3.40	catechin	291	139, 273
2	3.99	astragalin (kaempferol-3-glucoside)	449	257, 269, 287
3	5.88	6-gingerol	317	115, 117, 145
4	6.66	8-gingerol	345	115, 145
5	6.82	6-shogaol	277	94, 122, 137, 177

**Table 3:** Effects of DBZO on whole blood immune cell number in the blood of PM_2_._5_-exposed BALB/c mice.

	Unit: k/µL		
	NC	PM_2.5_	DBZO100
Total cells	1.6380 ± 0.6489 ^c^	4.0460 ± 0.7922 ^a^	2.8740 ± 1.0270 ^b^
Neutrophils	0.3464 ± 0.1538 ^c^	0.9327 ± 0.2122 ^a^	0.5902 ± 0.2009 ^b^
Lymphocytes	1.2477 ± 0.5594 ^c^	2.8743 ± 0.6679 ^a^	2.2140 ± 0.8076 ^b^
Monocytes	0.0298 ± 0.0158 ^a^	0.0396 ± 0.0166 ^a^	0.0402 ± 0.0242 ^a^
Eosinophils	0.0078 ± 0.0129 ^b^	0.1955 ± 0.1562 ^a^	0.0258 ± 0.0093 ^b^
Basophils	0.0062 ± 0.0057 ^a^	0.0040 ± 0.0055 ^a^	0.0037 ± 0.0051 ^a^

The results are presented as mean ± SD (*n* = 5). Data were statistically considered at *p* < 0.05, and different small letters represent the statistical differences.

## Data Availability

The data presented in this study are available on request from the corresponding author.

## Supplementary Materials

The following supporting information can be downloaded at: https://www.mdpi.com/article/10.3390/antiox13121572/s1, Figure S1: Effects of PM_2.5_ exposure on cell viability in A549 cells. The cells were treated with PM_2.5_ at concentrations of 0, 25, 50, 100, and 200 μg/mL for 4, 12, 24, and 48 h; Figure S2: Effects of PM_2.5_ exposure on apoptosis-related protein expression level in A549 cells. Western blot image (A) and protein expression level of cleaved caspase-3 (B); Figure S3: 50% ethanol extract of *Dioscorea bulbifera* of UPLC-Q/TOF-MS chromatogram (A) and MS fragments chromatogram (B).
