# Supplementary material for: A Combined Extract from Dioscorea bulbifera and Zingiber officinale Mitigates PM2.5-Induced Respiratory Damage by NF-κB/TGF-β1 Pathway
Source: Antioxidants (Basel). 2024 Dec 20;13(12):1572. doi: 10.3390/antiox13121572 (PMC11673267; doi:10.3390/antiox13121572)
Supplement: Supplementary file 1 [file antioxidants-13-01572-s001.zip › antioxidants-3342815-supplementary.pdf]

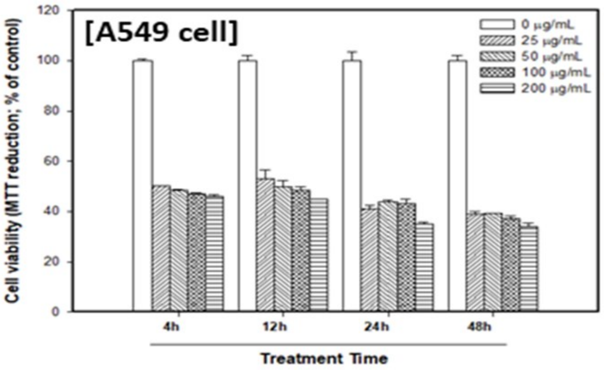

Figure S1. Effects of $PM_{2.5}$ exposure on cell viability in A549 cells. The cells were treated with $PM_{2.5}$ at oncentrations of 0, 25, 50, 100, and 200 μg/mL for 4, 12, 24, and 48 h. Result shown means ± SD ($n = 5$).

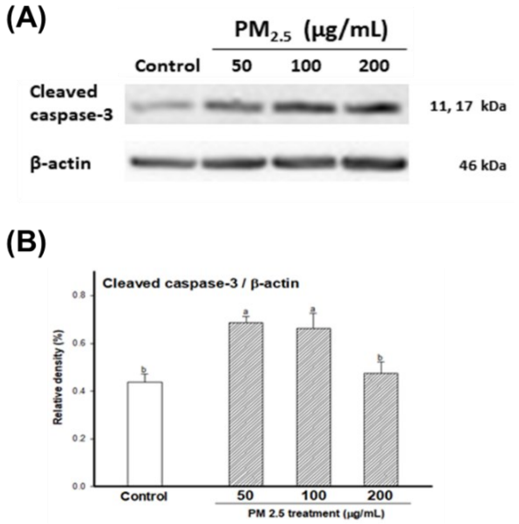

Figure S2. Effects of $PM_{2.5}$ exposure on apoptosis-related protein expression level in A549 cells. Western blot image (**A**) and protein expression level of cleaved caspase-3 (**B**). Result shown means ± SD ($n = 3$). Data were statistically considered at $p < 0.05$, and different small letters represent statistical differences.

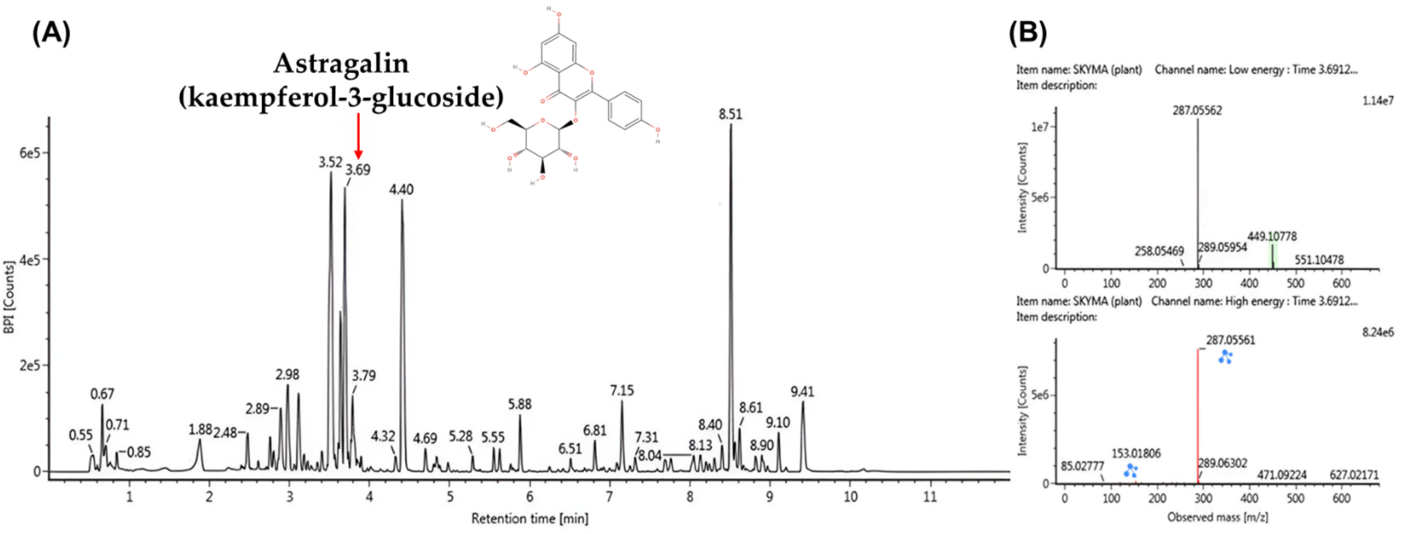

Figure S3. 50% ethanol extract of *Dioscorea bulbifera* of UPLC-Q/TOF-MS chromatogram (**A**) and MS fragments chromatogram (**B**).
